# Linkage to care and treatment outcomes for patients diagnosed with drug-susceptible tuberculosis using Xpert MTB/RIF assay in Thaba-Tseka district in Lesotho

**DOI:** 10.1016/j.ijregi.2022.08.010

**Published:** 2022-08-30

**Authors:** Nteseng Mabote, Meseret Mamo, Bienvenu Nsakala, Samson Lanje, Ntumwa R. Mwanawabene, Bulemba Katende

**Affiliations:** a Department of Public Health, Texila American University, Georgetown, Guyana; b Ministry of Health, Maseru, Lesotho; c Department of Epidemiology and Biostatistics, Stellenbosch University, Tygerberg, South Africa; d SolidarMed, Partner in Health, Thaba-Tseka, Lesotho; e Elisabeth Glaser Pediatric AIDS Foundation, Maseru, Lesotho

**Keywords:** Xpert MTB/RIF, Tuberculosis, Linkage to care, COVID-19, Lesotho

## Abstract

**Objectives:**

To evaluate linkage to care and treatment outcomes of patients with tuberculosis (TB) confirmed by Xpert MTB/RIF assay in Thaba-Tseka district, Lesotho.

**Design:**

This was a retrospective cohort study of adult patients diagnosed with drug-susceptible TB using the Xpert MTB/RIF assay at two laboratories in Thaba-Tseka district from January 2016 to December 2020.

**Results:**

Six hundred and fifty-five eligible participants were identified for inclusion in this study. Their median age was 40 [interquartile range (IQR) 32–54] years, and 468 (71.45%) were male. Evidence of linkage to care was found for 459 (70.08%) participants, but there was no documentation on treatment initiation for 196 (29.92%) participants. The median time to treatment initiation was 0 days (same-day initiation) (IQR 0–4) and the treatment success rate was 86%. Treatment success was associated with negative sputum smear results after 2, 5 and 6 months (χ^2^, *P*<0.001). The overall mortality rate was 10%, with no trend of mortality reduction.

**Conclusion:**

There is a need to address the issue of linkage to care of patients diagnosed with TB in Thaba-Tseka district. Efforts should be made to reduce TB mortality in line with the World Health Organization's ‘End TB strategy’ target.

## Highlights

• Patients with tuberculosis (TB) with evidence of linkage to care is not optimal in Thaba-Tseka district.
• Same-day TB treatment initiation is feasible for facilities with onsite GeneXpert services.
• The TB death rate in Thaba-Tseka district does not align with the World Health Organization's End TB strategy target.

## Introduction

Although the Xpert MTB/RIF assay is implemented worldwide for the diagnosis of tuberculosis (TB), the gap in case identification remains wide, especially in low- and middle-income countries (Albert et al., 2016). In 2020, the World Health Organization (WHO) estimated the number of new TB infections at 9.9 million; however, only 5.8 million TB cases were diagnosed and reported, while 4.1 million were missed (World Health Organization, 2021). Missed TB cases are left untreated, potentially spreading *Mycobacterium tuberculosis* in the community.

Lesotho has the highest incidence of TB in the world, estimated at 650 cases per 100,000 per year, and low treatment coverage that was estimated at 55% in 2019 before the coronavirus disease 2019 (COVID-19) pandemic and reduced to 33% in 2020 with the emergence of COVID-19 (Lesotho Ministry of Health, 2020; World Health Organization, 2020, 2021). A study analysing the Lesotho TB diagnostic network reported a national linkage to care rate of 82%, with 2806 of the 3435 patients diagnosed with TB in 2016 initiated on treatment; the remaining 18% of patients could not be traced (Albert et al., 2020). Another study assessing the impact of GeneXpert implementation on the diagnosis, treatment initiation and treatment outcomes of patients with rifampicin-resistant TB reported a linkage to care rate of 61% amongst patients diagnosed between 2014 and 2016; it is likely that the remaining 39% of patients never initiated drug-resistant treatment (Katende et al., 2020).

Thaba-Tseka is one of 10 districts in the Kingdom of Lesotho, situated in the mountainous north-eastern part of the country, dominated by difficult terrain and harsh weather. Thaba-Tseka district has an estimated population of 135,347, of which 80% reside in remote rural areas (Lesotho Bureau of Statistics, 2020). The health facilities in Thaba-Tseka district are, in most cases, not easily accessible, and patients have to walk for many hours to access basic healthcare services. Studies in similar settings report challenges with GeneXpert implementation, varying from delayed diagnosis to delayed treatment initiation (Iruedo et al., 2017; Nalugwa et al., 2020).

This study aimed to evaluate the linkage to care rate of patients diagnosed with drug-susceptible TB using the Xpert MTB/RIF assay in Thaba-Tseka district, given its specific terrain. In addition, we also assessed factors associated with treatment outcomes in these patients, and evaluate the impact of the COVID-19 pandemic on TB case notification.

## Methods

### Study design

A 5-year retrospective cohort study was undertaken of all adult patients diagnosed with rifampicin-susceptible tuberculosis using the GeneXpert assay at the two hospitals with laboratories offering Xpert MTB/RIF assay services in Thaba-Tseka district, Lesotho from 1 January 2016 to 31 December 2020.

### Settings

**Figure 1:**
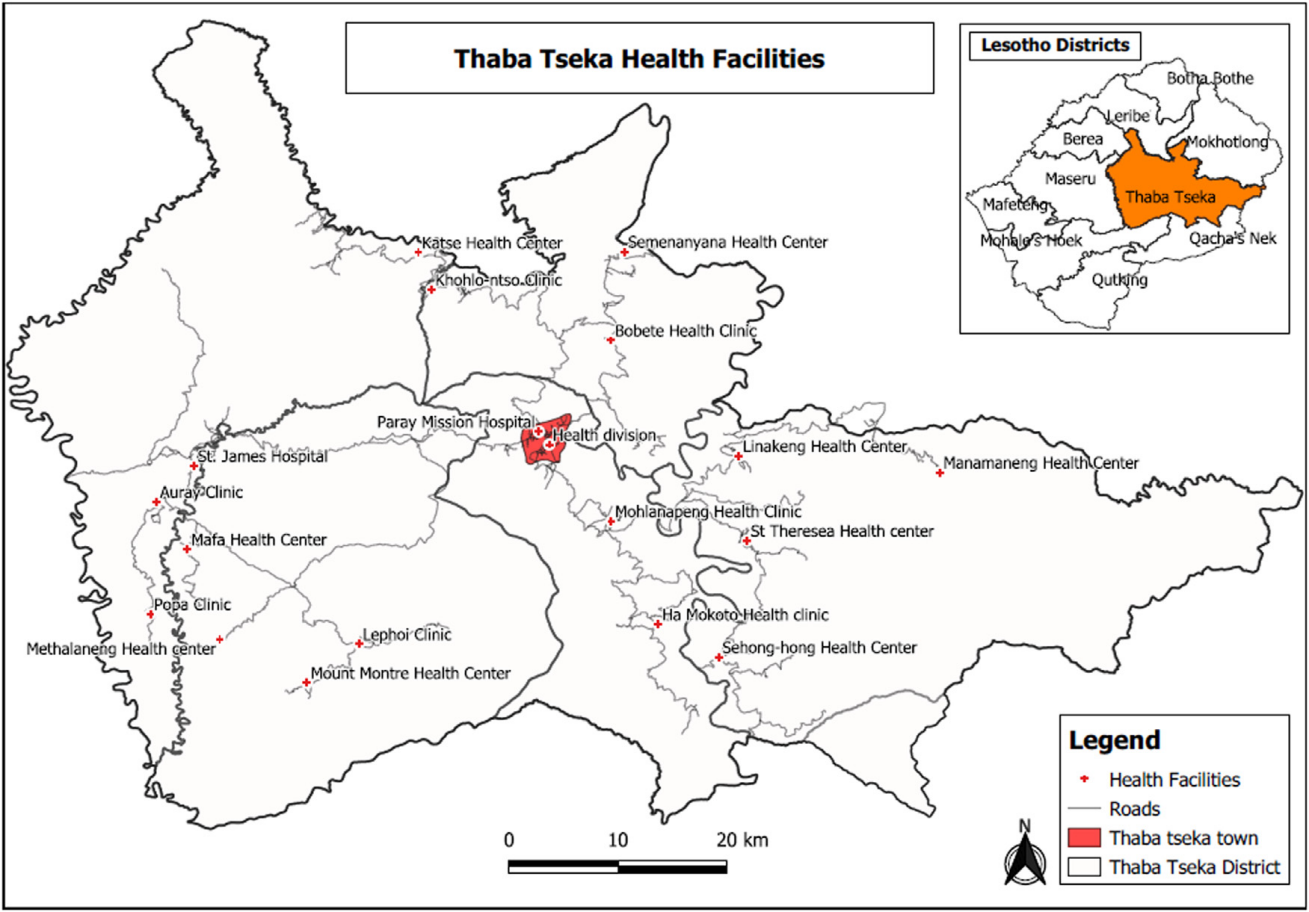
Map of Thaba-Tseka district showing all the health centres and hospitals included in the study.

Thaba-Tseka district is located in the north-east of Lesotho, and has many hard-to-reach areas. However, the model of delivery of health services is still mainly through fixed facilities, which includes 17 health centres (health clinics) and two hospitals, both owned by the Christian Health Association of Lesotho. Figure 1 shows all the health facilities in Thaba-Tseka district.

### Thaba-Tseka TB diagnostic network

During the study period, three laboratories provided Xpert MTB/RIF assay services in Thaba-Tseka district. Patients screened for TB submit sputum specimens to the health facilities if they present any TB symptoms, as stated in the Lesotho national guidelines for drug-susceptible TB (Lesotho Ministry of Health, 2019). The specimens are then transported by the requesting health facility to a laboratory for diagnostic testing.

### Study sites

All 19 health facilities – 17 health centres (Popa, Lephoi, Mont Marte, Auray, Katse, Khohlontso, Health Division, Mokoto, Mohlanapeng, Linakeng, Bobete, Manamaneng, St Theresa, Sehonghong, Mafa, Methalaneg) and both hospitals (St James Hospital and Paray Mission Hospital) – in Thaba-Tseka district were included in this study.

The two hospitals have laboratories that offered Xpert MTB/RIF assay services to 16 of the 19 health facilities (including the hospitals) during the study period. Due to time and financial constraints, diagnostic data were not collected from the Partners in Health laboratory, which is located outside Thaba-Tseka district; three of the 19 health facilities in Thaba-Tseka district sent their samples to this laboratory for TB testing during the study period.

### Data collection

A list of patients with positive GeneXpert results from 1 January 2016 to 31 December 2020 was generated from the two laboratories in Thaba-Tseka district offering Xpert MTB/RIF assay services, located at Paray Mission Hospital and St. James Hospital. Participants aged ≥18 years with a positive Xpert MTB/RIF result and susceptible and/or indeterminate rifampicin result were enrolled consecutively into the study. All eligible participants identified by Xpert MTB/RIF assay were then tracked at all the district health facilities to ascertain linkage to care.

The following information was collected from the GeneXpert facilities: patient demographics (name, age, gender); Xpert MTB/RIF assay and rifampicin susceptibility results; date of availability of Xpert MTB/RIF result; and referring health facility. These data was generated from the DISA software that is directly linked to the GeneXpert system.

At health centre or hospital level, the participants’ name, age and gender were used as they appeared in the TB treatment register to match the participants on the list obtained from the GeneXpert facilities. As participants could be initiated on treatment at a health facility other than the health facility that sent their sample for TB testing, study participants were tracked at all 19 health facilities in Thaba-Tseka district. A study participant was considered to be linked to care if it was possible to match him/her in the TB register using the above-mentioned strategy.

For participants linked to care, the following information was collected from the TB treatment register: date of treatment initiation; occupation/high-risk category; history of previous TB treatment; human immunodeficiency virus (HIV) status; use of antiretroviral therapy; chest X-ray result; sputum acid-fast bacilli test result after 2, 5 and 6 months; and treatment outcome.

Time to treatment initiation was defined as the time from the date of availability of the GeneXpert result to the date of treatment initiation. The WHO definitions of treatment outcomes were used: treatment completed, cured, treatment failed, died, not evaluated, lost to follow-up, and treatment success (Lesotho Ministry of Health, 2019). Patients classed as ‘not evaluated’ were excluded from the treatment outcomes analysis.

### Data analysis

STATA Version 14.2 (STATA Corp, College Station, TX, USA) was used for data analysis. *P*≤ was considered to indicate significance. The demographics of study participants are presented using frequency tables, mean, standard deviation and 95% confidence interval (CI) for normally distributed data, and median and interquartile range (IQR) for skewed data. Chi-squared test and Fisher's exact test were used to assess the association of treatment outcome with sputum conversion, gender, occupation, history of previous TB treatment, HIV status and referring health facility.

### Ethical approval

This study was conducted in accordance with Lesotho regulations for medical research. Before implementation of the study, the study protocol was submitted to Lesotho Ministry of Health Ethics Committee for approval (Reference No. 02-2222). The Ethics Committee granted permission for patient records to be reviewed without informed consent as there was no direct contact with the study participants.

## Results

In total, 672 participants with a positive Xpert MTB/RIF result were identified from the two laboratories providing GeneXpert assay services for TB diagnosis in Thaba-Tseka district. Of these, 17 patients were excluded from the study because they did not meet the study entry criteria: nine patients were aged <18 years, and eight patients had a positive Xpert MTB/RIF result with rifampicin resistance. As such, 655 patients were enrolled in the study.

The TB treatment registers of each of the health facilities in Thaba-Tseka district were checked to establish linkage to care for the 655 eligible participants. In total, of the 655 patients with positive Xpert MTB/RIF results, 459 (70.08%) had linkage to care; linkage to care could not be ascertained for the remaining 196 (29.92%) participants. There was evidence of linkage to care for ≥60% of patients with positive Xpert MTB/RIF results for all health centres and hospitals, apart from Popa health centre (56%) (Table 1). Linkage to care improved over time, with the best linkage rates observed in 2019 and 2020. The COVID-19 pandemic did not seem to impact the linkage to care rate (Table 2). No differences in age (χ^2^, *P*=0.77) and gender (χ^2^, *P*= 0.06) were found between participants linked to care and those without evidence of care.

**Table 1:** Linkage to care rates, time to treatment initiation, treatment success and death rates by health facility

Facility	Total Xpert positive *n*(%=*n*/N*100)	Total Xpert positive linked to care *k*(%=*k*/n*100)	Time to treatment initiation, median (IQR)	Treatment success rate *p*(%=*p*/m*100)	Death rate *q*(%=*q*/m*100)
Auray	13 (1.98)	10 (76.92)	1 (0–3)	9 (100)	0 (0)
Health Division	24 (3.66)	15 (62.5)	2 (1–5)	10 (76.92)	1 (7.69)
Katse	21 (3.21)	18 (85.71)	5.5 (1–8)	16 (88.89)	2 (11.11)
Khohlontso	7 (1.07)	5 (71.42)	4 (1–7)	5 (100)	0 (0)
Lephoi	15 (2.29	10 (66.66)	3.5 (0–6)	6 (66.67)	3 (33.33)
Linakeng	9 (1.37)	9 (100)	6 (3–9)	7 (77.78)	2 (22.22)
Mafa	11 (1.68)	7 (63.64	2 (2–4)	7 (100)	0 (0)
Mohlanapeng	19 (2.90)	15 (78.94)	4 (2–7)	12 (85.71)	0 (0)
Mokoto	20 (3.05)	19 (95)	3 (3–6)	16 (88.89)	1 (5.56)
Montmartre	19 (2.90)	16 (84.21)	1 (0.5–9.5)	15 (93.75)	1 (6.25)
Paray Mission Hospital	349 (53.28)	224 (64.18)	0 (0–0)	191 (86.82)	21 (9.55)
Popa	16 (2.44)	9 (56.25)	17 (9–19)	8 (88.89)	1 (11.11)
Sehonghong	20 (3.05)	18 (90)	4.5 (2–7)	15 (93.75)	1 (6.25)
Semenenyane	16 (2.44)	12 (75)	10 (7–13.5)	11 (91.67)	0 (0)
St. James Hospital	66 (10.08)	46 (69.70	0 (0–0)	38 (84.44)	6 (13.33)
St. Theresa	30 (4.58)	26 (86.67)	6 (2–8)	18 (72.00)	5 (20)

IQR, interquartile range.

*N*, total number of participants eligible for the study; *n*, total number of participants eligible for the study per health facility; *k*, total number of participants eligible for the study linked to care; *m*, total number of participants with a treatment outcome per facility, participants with treatment outcome ‘not evaluated’ were excluded; *p*, total number of participants with treatment success per facility; q, total number of participants who died.

**Table 2:** Linkage to care rates and death rates by year of diagnosis

Year of diagnosis	Total Xpert positive	Total Xpert positive linked to care	Percentage Xpert positive linked to care (%)	Total eligible for treatment outcome analysis	Total died *n* (%)
2016	198	117	59.09	113	13 (11.5)
2017	131	72	54.96	71	9 (12.67)
2018	135	105	77.77	103	10 (9.71)
2019	112	97	86.61	95	6 (6.32)
2020	79	68	86.07	63	6 (9.52)
Overall	655	459	70.07	445	44 (9.89)

The median age of all study participants was 40 (IQR 32–54) years and 468 (71.45) were male. For participants linked to care, 95 (20.70%) had a previous history of TB treatment, 294 (64.05%) had a positive HIV status, and 293 (99.66%) of those with a positive HIV status were on antiretroviral therapy. Table 3 summarizes the baseline characteristics of the study participants linked to care.

**Table 3:** Baseline characteristics of study participants

	Total *n* (%)
Age group (*n*=655)	
18–24	48 (7.3%)
25–34	168 (25.7%)
35–44	184 (28.1%)
45–54	97 (14.8%)
55–64	78 (11.9%)
≥65	80 (12.21%)
Gender (*n*=655)	
Male	468 (71.45%)
Female	187 (28.55%)
Occupation (*n*=459)	
Healthcare worker	1 (0.22)
Mine/ex-mine worker	183 (39.87)
Factory worker	11 (2.40)
Public transport operator	7 (1.53)
Household (current mine/ex-mine worker)	18 (3.92)
Prisoner	6 (1.31)
Unemployed	118 (25.71)
Farmer	31 (6.75)
Self-employed	11 (2.40)
Other (builder, student, domestic worker, housewife)	69 (15.03)
Missing	4 (0.87)
History of previous tuberculosis (*n*=459)	
Yes	95 (20.70%)
No	361(78.64)
Missing	3 (0.65)
Human immunodeficiency virus status (*n*=459)	
Negative	159 (34.64%)
Positive	294 (64.05%)
Unknown	6 (1.31%)
Use of antiretroviral therapy (*n*=294)	
Yes	293 (99.66%)
No	1 (0.34%)

In the participants with linkage to care, the median time to treatment initiation was 0 (IQR 0–4) days (same-day treatment initiation). Popa and Semenenyane health centres had the longest times to treatment initiation [median 17 (IQR 9–19) days and 10 (IQR 7–13.5) days, respectively], and the two hospitals (Paray Mission Hospital and St James Hospital) had the shortest times to treatment initiation [median 0 (IQR 0–0) days for both].

Regarding treatment outcomes, 445 (96.94%) of the 459 patients with linkage to care were analysed (see Figure 1). Of these, 384 (86%) had treatment success, 10 (2.25%) were lost to follow-up, 7(1.6%) had treatment failure and 44 (10%) died. The highest mortality rate was seen in 2017, and the lowest mortality rate was seen in 2019; there was no specific trend in mortality (see Table 2).

Treatment success was associated with a negative smear result after 2, 5 and 6 months (χ^2^, *P*<0.001). No associations were found between treatment outcome and gender (χ^2^, *P*=0.70), occupation (χ^2^, *P*=0.35), history of previous TB (Fisher's exact test, *P*=0.11), HIV status (Fisher's exact test, *P*=0.24), and referring health facility (χ^2^, *P*=0.18).

In 2020, when the first case of COVID-19 occurred in Lesotho and a hard lockdown was implemented, only 79 (12.06%) cases of drug-susceptible TB were identified using the GeneXpert assay. This was the lowest number from the 5 years studied, during which a total of 655 cases were identified. When compared with 2019, there was a 29.5% reduction in the identification of cases of drug-susceptible TB in 2020.

Figure 2

**Figure 2:**
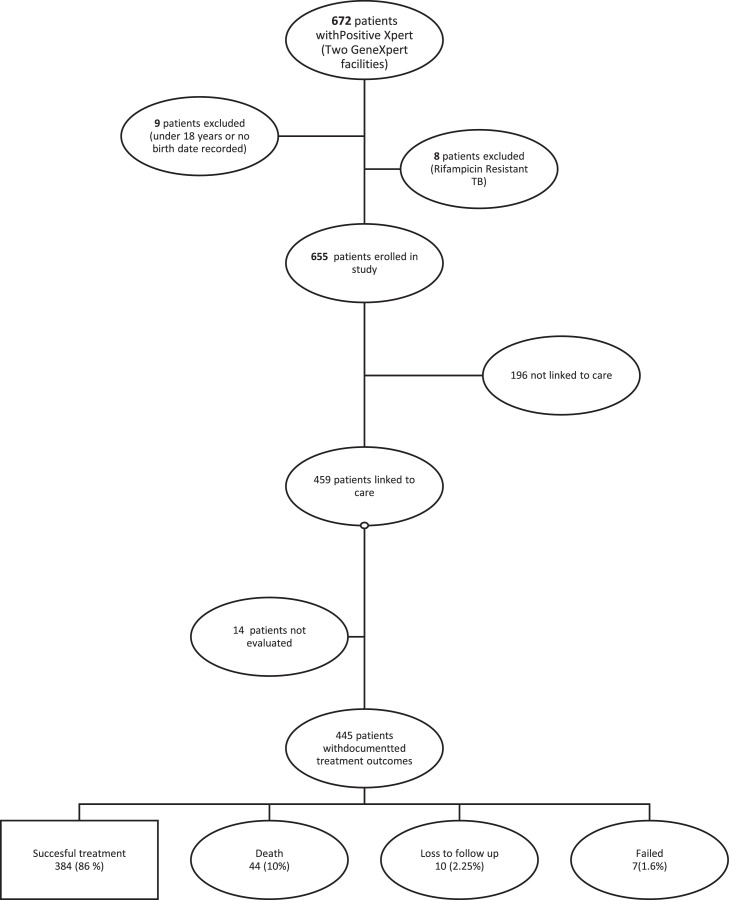
Study flowchart.

## Discussion

Over a decade since adoption of the Xpert MTB/RIF assay as a diagnostic tool for TB, and 7 years since the introduction of the WHO ‘End TB strategy’, this study evaluated the linkage to care of patients diagnosed with TB, a critical parameter for TB incidence and mortality.

This study found a linkage to care rate of 70.08% for patients diagnosed with drug-susceptible TB using the Xpert MTB/RIF assay at the two facilities offering Xpert MTB/RIF assay services in Thaba-Tseka district. As such, 29.92% of patients diagnosed with TB did not have any evidence of linkage to care in Thaba-Tseka district between 2016 and 2020. The linkage to care rate was 54.92% in 2017, and increased to 86% in 2019 and 2020.

The low linkage to care rate observed in this study is similar to rates reported in various studies in Southern Africa (Maraba et al., 2018; Bassett et al., 2019; Vanqa et al., 2021). A multi-centre study conducted in South Africa and Uganda reported a linkage to care rate of 55% (12/22) amongst HIV-positive patients diagnosed with active drug-susceptible TB, while a study in Malawi reported a linkage to care rate of 40% (23/58) amongst hospitalized patients diagnosed with TB in Lilongwe (Chawla et al., 2016; Shapiro et al., 2018). In Lesotho, a study assessing treatment initiation of patients diagnosed with rifampicin-resistant TB using the GeneXpert assay reported a linkage to care rate of 60.6% (314/528) (Katende et al., 2020).

The median time to treatment initiation in this study was 0 (IQR 0–4) days. Most previous studies did not report time to treatment initiation in patients diagnosed with drug-susceptible TB. However, this study found a shorter median time to treatment initiation compared with times reported for patients diagnosed with rifampicin-resistant TB using the Xpert MTB/RIF assay in South Africa [Johannesburg 13 (IQR 7–28) days, Eastern Cape 15 (IQR 8–23) days, Cape Town 8 (IQR 5–25) days] and Lesotho [12 (IQR 7–19) days] (Cox et al., 2017; Iruedo et al., 2017; Evans et al., 2018; Katende et al., 2020).

This study found an overall treatment success rate of 86% amongst patients diagnosed with drug-susceptible TB in Thaba-Tseka district, and a TB mortality rate of 10%. The treatment success rate is comparable to the global treatment success rate of ≥85% reported in patients initiated on first-line TB treatment since 2017 by WHO, and is better than the success rate of 76% reported in a review of patients with bacteriologically confirmed TB initiated on first-line TB treatment in Sub-Saharan Africa (World Health Organization, 2018, 2019, 2020, 2021). In the latter review, the findings may have been affected by the inclusion of studies conducted before the endorsement of Xpert MTB/RIF technology by WHO in 2010; these studies may have enrolled patients with rifampicin-resistant TB on first-line TB treatment (Izudi et al., 2019). However, the TB treatment success rate in Thaba-Tseka district does not meet the WHO target of ≥90% (World Health Organization, 2015).

The TB mortality rate remained relatively constant from 2016 to 2020 in this study, with no specific trend. A South African study reported a slightly low cumulative mortality rate of 8.6% for adult patients who initiated drug-susceptible TB treatment (Osman et al., 2021). The TB mortality rate in Thaba-Tseka district does not meet the 2020 ‘End TB strategy’ target of a 35% reduction in mortality rate from 2015. Moreover, the trend in TB mortality from 2016 does not suggest that the 2025 ‘End TB strategy’ target of a 75% reduction in mortality rate will be met in Thaba-Tseka district.

To the authors’ knowledge, this study is the first of its kind conducted in a district such as Thaba-Tseka where the terrain makes health service delivery very challenging. The inclusion of a large number of health facilities plus a large sample size should have reduced the risk of bias considerably. However, the study had the following limitations: exclusion of diagnostic data from one laboratory that served three health centres; use of routine data; and inability to trace patients throughout Lesotho.

Although the authors made significant efforts to trace all the study participants at all health facilities in Thaba-Tseka district, study participants with no evidence of care within the district may have initiated treatment in health facilities outside of Thaba-Tseka district or even in neighbouring South Africa. As most of the study participants were of working age, they may have sought treatment in districts where they could still work while taking their medication. Some participants may have changed their names between accessing the GeneXpert facility and the treating health facility, making it impossible to match them. Some patients may have died before initiating treatment.

In conclusion, Thaba-Tseka district still faces challenges linking to care patients with bacteriologically confirmed TB. Although the treatment success rate is close to the WHO target, the TB mortality rate remains high. Thaba-Tseka district should continue to implement the strategies that improved the linkage to care rates in 2019 and 2020, in combination with other evidence-based strategies, to improve the linkage to care rate and treatment success rate, and reduce the TB mortality rate in the district. Involving the laboratories that provide Xpert MTB/RIF assay services in the linkage to care of patients they diagnose can play a crucial role in reducing the gap between the number of confirmed TB patients and the number of TB patients who initiate treatment.

## Appendix Supplementary materials

Image, application 1
